# Tumor mutation burden testing: a survey of the International Quality Network for Pathology (IQN Path)

**DOI:** 10.1007/s00428-021-03093-7

**Published:** 2021-04-15

**Authors:** Francesca Fenizia, Nicola Wolstenholme, Jennifer A. Fairley, Etienne Rouleau, Melanie H. Cheetham, Martin P. Horan, Emina Torlakovic, Benjamin Besse, Raed Al Dieri, Dina G. Tiniakos, Zandra C. Deans, Simon J. Patton, Nicola Normanno

**Affiliations:** 1 Cell Biology and Biotherapy Unit, Istituto Nazionale Tumori - IRCCS - “Fondazione G. Pascale”, Via Mariano Semola, 80131 Napoli, Italy; 2 EMQN CIC, c/o Trustech, 6th Floor, Citilabs 1.0, Nelson Street, Manchester, M13 9NQ UK; 3 Genomics Quality Assessment, Department of Laboratory Medicine, Royal Infirmary of Edinburgh, Little France Crescent, Edinburgh, EH16 4SA UK; 4 Department of Medical Biology and Pathology, Gustave Roussy, Cancer Genetics Laboratory, Gustave Roussy, 94800 Villejuif, France; 5 RCPAQAP Molecular Genetics, St Leonard’s, Sydney Australia; 6 Department of Pathology and Laboratory Medicine, Royal University Hospital, Saskatchewan Health Authority, Saskatoon, Saskatchewan Canada; 7 College of Medicine, University of Saskatchewan, Saskatoon, Saskatchewan Canada; 8 Department of Medical Oncology, Gustave Roussy University Hospital, 114 rue Edouard Vaillant, 94805 Villejuif, France; 9 European Society of Pathology, Brussels, Belgium; 10 Department of Pathology, Aretaieion Hospital, National and Kapodistrian University of Athens, Athens, Greece; 11 Translational & Clinical Research Institute, Faculty of Medical Sciences, Newcastle University, Newcastle upon Tyne, UK

**Keywords:** Tumour mutation burden, Next-generation sequencing, Immune-checkpoint inhibitors, Biomarkers

## Abstract

While tumour mutation burden (TMB) is emerging as a possible biomarker for immune-checkpoint inhibitors (ICI), methods for testing have not been standardised as yet. In April 2019, the International Quality Network for Pathology (IQN Path) launched a survey to assess the current practice of TMB testing. Of the 127 laboratories that replied, 69 (54.3%) had already introduced TMB analysis for research purposes and/or clinical applications. Fifty laboratories (72.5%) used targeted sequencing, although a number of different panels were employed. Most laboratories tested formalin-fixed paraffin-embedded material (94.2%), while 18/69 (26%) tested also cell-free DNA. Fifty-five laboratories used both single nucleotide variants and indels for TMB calculation; 20 centers included only non-synonymous variants. In conclusion, the data from this survey indicate that multiple global laboratories were capable of rapidly introducing routine clinical TMB testing. However, the variability of testing methods raises concerns about the reproducibility of results among centers.

**Supplementary Information:**

The online version contains supplementary material available at 10.1007/s00428-021-03093-7.

## Introduction

Immune-checkpoint inhibitors (ICI) represent a major breakthrough for the treatment of cancer patients. However, the activity of ICI varies between tumour types and among patients carrying tumours with the same histology. These observations led to the search of biomarkers that might aid in the stratification of patients eligible for treatment with ICI. PD-L1 expression and microsatellite instability (MSI) demonstrated to be clinically useful biomarkers.

Tumour mutation burden (TMB) is emerging as a possible biomarker to predict patient responses to immunotherapy [6]. As such, clinical interest for TMB testing has rapidly grown. TMB is defined as the total number of somatic mutations per coding area of a tumour genome. There is large variability in mutation burden within tumour types, ranging from just a few to thousands of mutations [1]. Higher TMB has been shown to correlate with higher levels of neoantigens [2]. Therefore, measuring mutational load can act as a proxy for determining the number of neoantigens in a tumour.

TMB has been associated with response to ICI in multiple cancer types [4, 10, 12–14]. However, the results of clinical trials that explored TMB as a biomarker for ICI have not been consistent. While some studies showed a significant correlation between TMB values and outcome for patients treated with ICI, other trials failed to confirm such a correlation. Several factors may contribute to these inconsistent findings, including the complexity of the mechanisms regulating the anti-tumor immune response. Importantly, the Food and Drug Administration (FDA) recently approved pembrolizumab for treatment of TMB-high unresectable or metastatic solid tumours.

TMB can be estimated by whole-exome sequencing (WES) but this method is unrealistic on a large-scale clinical-diagnostic setting due to high cost and quantity of tissue needed. Studies have shown that mutational burden of the whole genome can be inferred from sequencing small panels consisting of a few hundred genes [5, 8, 11]. In this respect, different targeted sequencing panels for measurement of TMB have been employed in clinical trials of ICI treatment [3].

Although TMB was not yet approved as a biomarker by regulatory agencies, many laboratories world-wide began to offer TMB testing in 2019. Therefore, the International Quality Network for Pathology (IQN Path) launched a project aiming to harmonise TMB testing by supporting the organization of a pilot external quality assessment (EQA) scheme. Seven different EQA providers are participating in this project.

The first step of the IQN Path TMB project was to launch a survey to identify laboratories that were currently offering TMB testing. This paper summarises the results of the survey and offers an overview of current practices used for TMB testing across the world.

## Methods

Seven different EQA providers, under the umbrella of IQN Path, designed an online survey to be distributed among the participants of each provider. They were the Italian Association of Medical Oncology (AIOM), the European Molecular Genetics Quality Network (EMQN), the French EQA organization Gen&Tiss, Genomics Quality Assessment (GenQA), the European Society of Pathology (ESP) Foundation, the Canadian Immunohistochemistry Quality Control (cIQc) and the Royal College of Pathologists of Australasia Quality Assurance Programs (RCPAQAP).

The survey included groups of questions on the following: (i) the accreditation and participation in EQA schemes; (ii) the techniques available and the tests offered in molecular pathology; (iii) the scope of next-generation sequencing (NGS) and the NGS approaches used; (iv) the availability of TMB testing and the method used for this analysis.

The survey was opened at the end of April 2019 and closed in May 2019. The responses were analysed to understand current practices in the field of NGS and more specifically TMB testing, and will inform the design of a future EQA pilot scheme.

## Results

Completed survey responses were submitted by 127 laboratories. The geolocation of the centers that participated is shown in Supplementary Fig. 1. The majority of laboratories were accredited/certified (112/127, 88.2%), offered molecular pathology diagnostic tests (119/127, 93.7%) and routinely participated in EQA schemes (120/127, 94.5%).

**Fig. 1:**
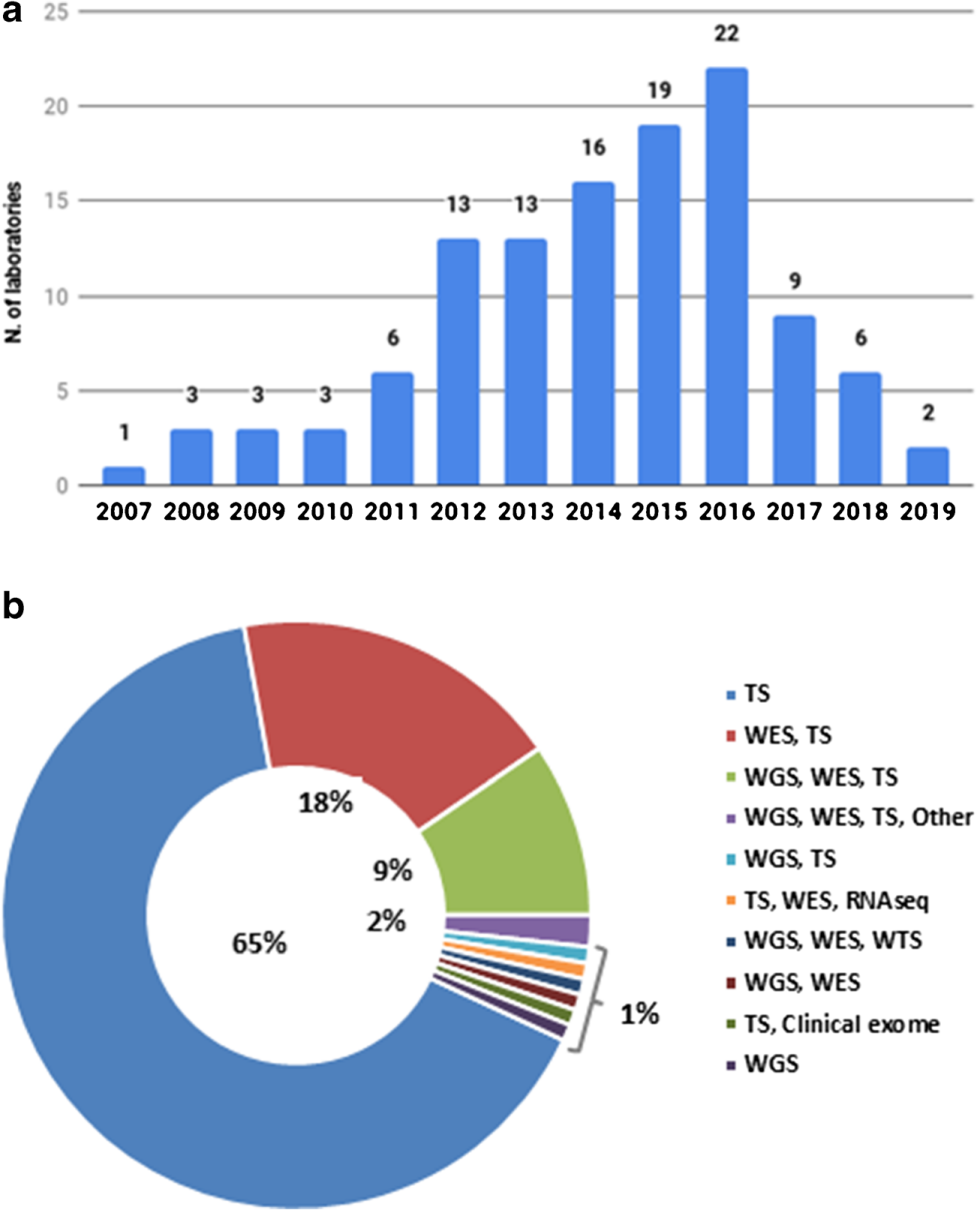
**a** Number of laboratories that introduced NGS since 2007. **b** NGS approaches used by the laboratories that participated to the survey. TS: targeted sequencing; WES: whole-exome sequencing; WGS: whole-genome sequencing; WTS: whole-transcriptome sequencing; RNAseq: RNA sequencing

Of the 127 laboratories that responded, 119 (93.7%) had NGS platforms and 117 (92.1%) used NGS for tumour genomic profiling. Few laboratories introduced NGS as a testing method before 2012 (Fig. 1a). Almost all centers employed targeted sequencing, while some also performed WES, whole-genome sequencing (WGS) and/or RNA sequencing (Fig. 1b). All laboratories had at least one Illumina or ThermoFisher NGS platform. Specifically, 48 laboratories used only Illumina platforms, 29 only ThermoFisher and 42 both Illumina and ThermoFisher sequencers. Eleven laboratories owned other NGS machines from different vendors, including 6 GeneReader (Qiagen), 3 BGISEQ (BGI Genomics) and 3 MGISEQ (MGI Tech Co.).

Sixty-nine laboratories (54.3%) had already introduced TMB analysis at the time of the survey, while 33 (26%) centers planned to introduce the test in their routine practice between the end of 2019 and the start of 2020. Twelve laboratories performed TMB testing for research purposes only, 13 for clinical applications only and 44 for both activities.

**Fig. 2:**
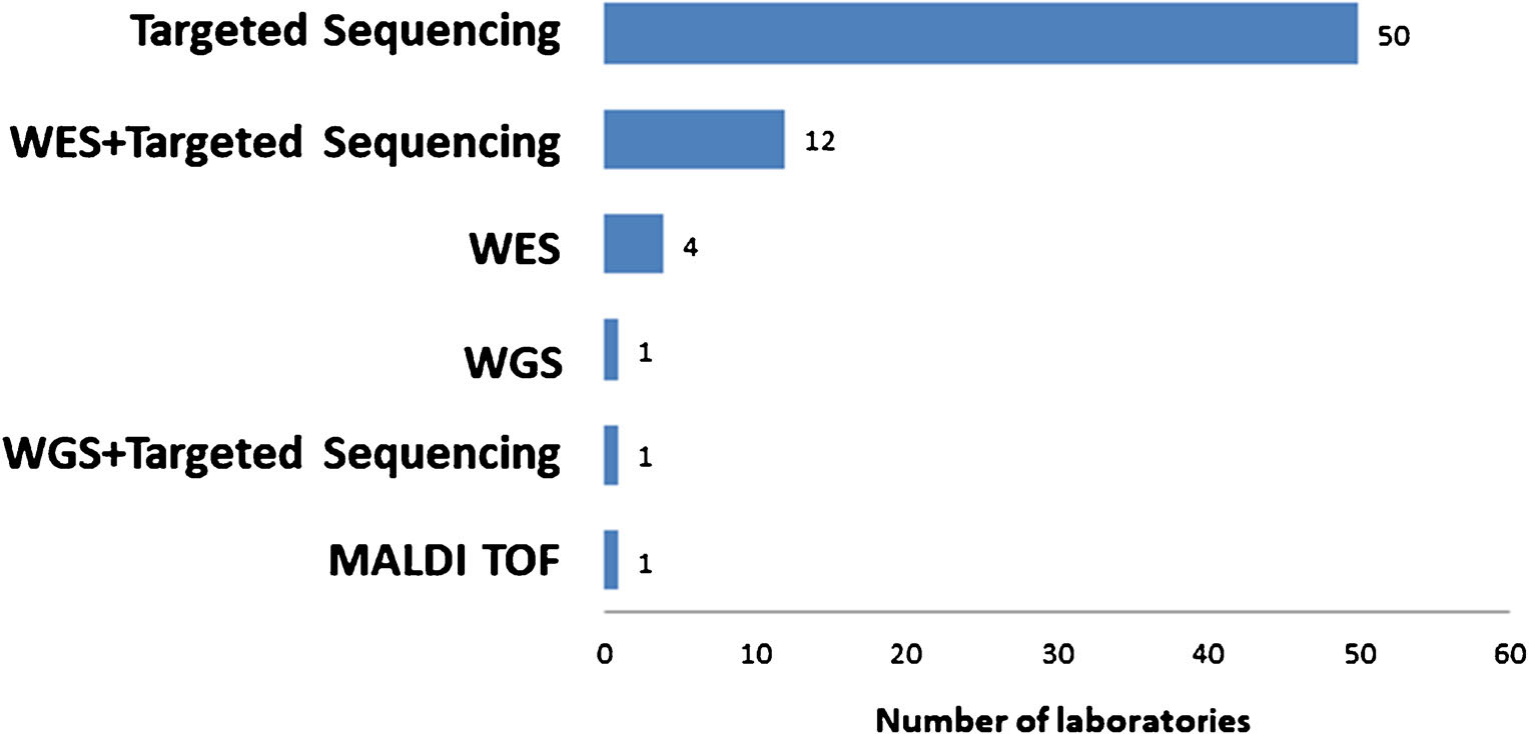
Methods used for TMB analyses by the laboratories that participated to the survey. WES: whole-exome sequencing; WGS: whole-genome sequencing

Fifty of 69 laboratories (72.5%) used targeted sequencing for TMB analysis (Fig. 2). Some centers employed WES plus targeted sequencing (12/69, 17.4%) or WES only (4/69, 5.8%). The techniques of WGS, WGS plus targeted sequencing or MALDI-TOF were rare. Laboratories used a number of different panels for TMB testing and six combined two different panels (Table 1). The most frequently used targeted sequencing panel was the Oncomine^TM^ Tumor Mutation Load. However, 18 centers developed their own custom panels.

**Table 1:** Targeted sequencing panels used for TMB testing

Panel	No. of laboratories
Oncomine^TM^ Tumor Mutation Load	21
Custom panels	18
TruSight^TM^ Oncology 500	6
TruSight^TM^ Oncology 500 + Oncomine^TM^ Tumor Mutation Load	3
Oncomine^TM^ Comprehensive Assay	2
Oncomine^TM^ (not specified)	2
QIAseq^TM^ Tumor Mutational Burden Panel	2
Oseq^TM^-T BGI	2
Oncomine^TM^ Tumor Mutation Load + Oncomine^TM^ Comprehensive Assay	1
TruSight^TM^ Oncology 500 + QIAseq^TM^ Tumor Mutational Burden Panel	1
Oncomine^TM^ Comprehensive Assay + TruSight^TM^ Oncology 500	1
NEOplus^TM^ V2 RUO	1
YyveOne^TM^ Plus	1
Avenio^TM^ Expanded ctDNA	1

The majority of laboratories utilised formalin-fixed paraffin-embedded (FFPE) material for TMB testing (65/69). Two laboratories used fresh/frozen tissue, one used liquid biopsy (i.e. cell-free DNA, cfDNA) and one both fresh/frozen tissue and liquid biopsy. Among the laboratories testing FFPE material, 22 also performed the TMB test on fresh/frozen tissue and 17 on liquid biopsy.

For TMB estimation, 55 laboratories (79.7%) calculated against both single nucleotide variants (SNVs) and indels, while 20 centers (29%) included only non-synonymous variants. Only 27/69 (39.1%) laboratories used a cut-off for the interpretation of TMB. However, several different types of cut-off were employed, based either on the literature, internal validation or validation against the FoundationOne test. The majority of laboratories tested tumours of different histology. However, almost every center analysed lung carcinomas. The number of TMB tests performed by the responding laboratories varied from 10 to 25,000 (Supplementary Figure 2).

## Discussion

The introduction of a new biomarker into routine clinical practice can be challenging for laboratories. This issue is even more relevant for TMB, which is a quantitative marker that requires appropriate NGS methodology and bioinformatics. To date, the optimal workflow for TMB testing and reporting has not yet been defined. Lack of such standardisation might be, in part, the reason for the conflicting results reported in clinical trials. In this respect, a recent study by the Quality In Pathology (QuIP) German organization on TMB testing revealed that misclassification of samples (high vs low TMB) occurred in approximately 25% of the cases [15].

Laboratory take-up of new biomarker testing generally tends to be impeded by cost, the requirement for specialised equipment or the need for further clinical validation. Quite surprisingly, over 50% of the laboratories that participated in this survey were offering TMB testing in April 2019, although this biomarker was not yet approved by regulatory agencies. This might suggest laboratories pay more attention to new biomarkers explored in clinical trials. In this respect, TMB testing was performed for clinical activities in the majority of laboratories (57/69), telling that clinicians’ demand for the TMB test is high. The recent FDA approval of pembrolizumab for treatment of TMB-high metastatic solid tumours will likely further increase the request for TMB testing. However, the laboratories that participated in this survey offered the TMB test mainly for lung cancer. This survey was conducted in 2019, after the publication of preliminary data suggesting an important role for TMB in identifying patients with non-small cell lung cancer (NSCLC) sensitive to treatment with a combination of ICIs [10]. At that time, oncologists started to request for TMB for NSCLC patients, and laboratories offered the test. However, TMB testing in NSCLC is not supported by the current ICIs approvals.

A variety of methods were used by the laboratories for TMB testing. The Oncomine^TM^ Tumor Mutation Load represented the most utilised panel. This is not a surprising finding, given that this panel was commercially available from late 2018, whereas other panels were launched just around the start of the study. However, many centers also used laboratory-developed techniques for TMB testing. Importantly, the type of mutations included in the TMB calculation (SNV and/or indels; synonymous and/or non-synonymous) and the cut-off used to interpret TMB results significantly varied among participating centers, potentially leading to different interpretations that may impact on the clinical management of the patient. These observations highlight again the need for standardisation of genomic biomarker testing and appropriate EQA schemes. In this respect, quite surprisingly 5.5% of laboratories declared that they did not routinely participated in EQA schemes. This finding probably reflects the fact that participation in EQA schemes is not mandatory in many countries, where laboratories often do not have a budget available for participation in these activities.

Surprisingly, 19 laboratories reported TMB testing using liquid biopsy (or cfDNA). When this survey started, very little data was available on the use of cfDNA for TMB testing [9]. Liquid biopsies are challenging for TMB testing owing to the limited amount of cfDNA that can be isolated from peripheral blood and the very low allelic frequency of variants found in the blood [7]. In this respect, only one laboratory used a panel specific for cfDNA testing, the Avenio^TM^ Expanded ctDNA. Unfortunately, laboratories participating to this survey did not provide any comparative data between tissue and liquid biopsy TMB testing, which are definitely needed to assess the analytical performance of liquid biopsy.

In conclusion, this survey highlights that many laboratories were able to introduce TMB testing quickly and effectively. The variability of methods used for testing raises issues on the reproducibility of results among different centers. An EQA program will contribute to the standardisation of TMB testing.

## Supplementary information

Supplementary Figure 1. Geolocation of the centers that participated to the survey (PNG 593 kb)

High resolution image (TIF 114 kb)

Supplementary Figure 2. Number of TMB tests performed by the responding laboratories. * = some laboratories did not disclose the number of TMB tests that they performed (PNG 162 kb)

High resolution image (TIF 124 kb)
