# Supplementary figures and images for: Tumor mutation burden testing: a survey of the International Quality Network for Pathology (IQN Path)
Source: Virchows Arch. 2021 Apr 15;479(6):1067–72. doi: 10.1007/s00428-021-03093-7 (PMC8724102; doi:10.1007/s00428-021-03093-7)

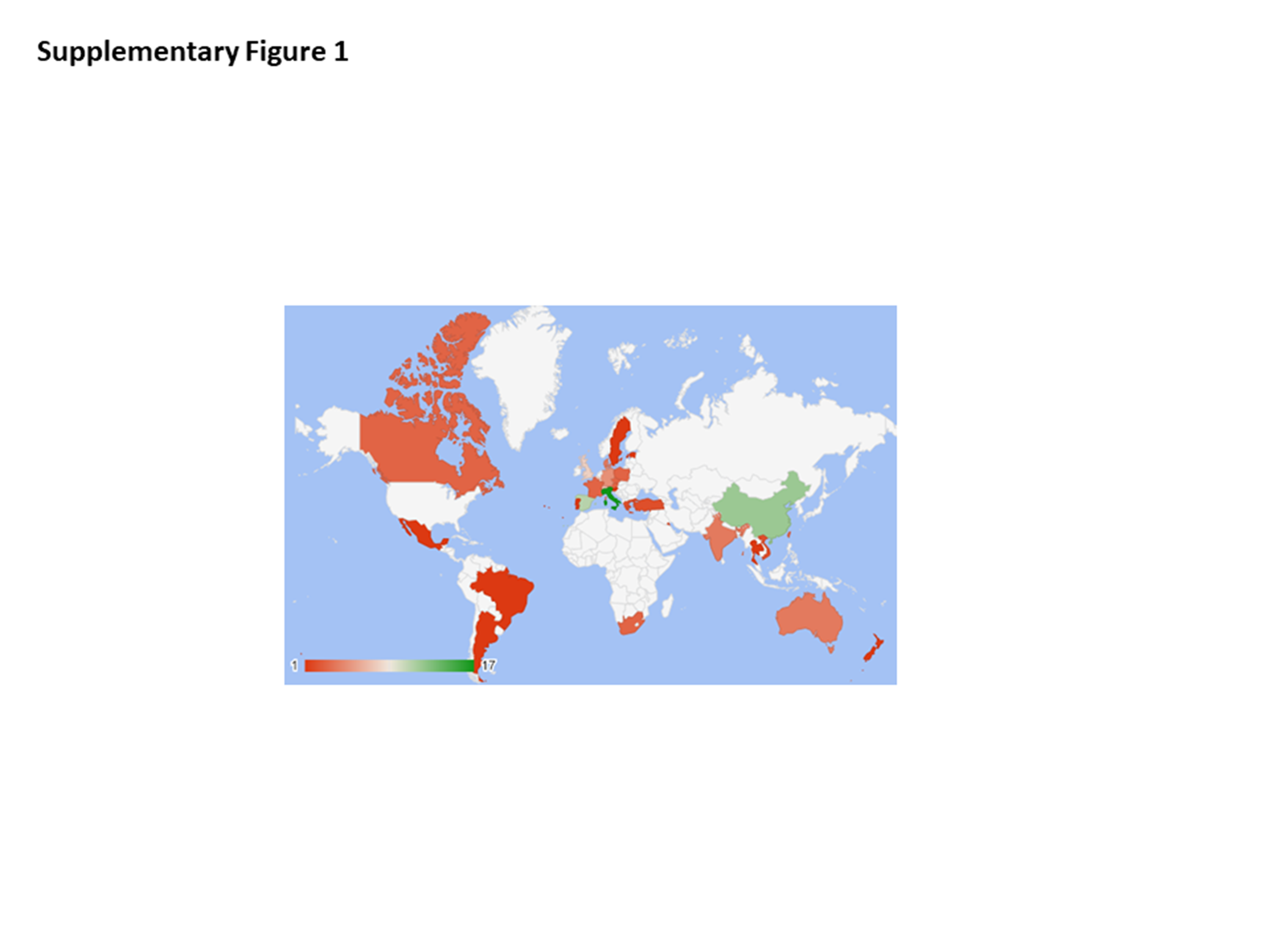

Supplement: Supplementary file 1 — Geolocation of the centers that participated to the survey (PNG 593 kb) [file 428_2021_3093_Fig3_ESM.png]

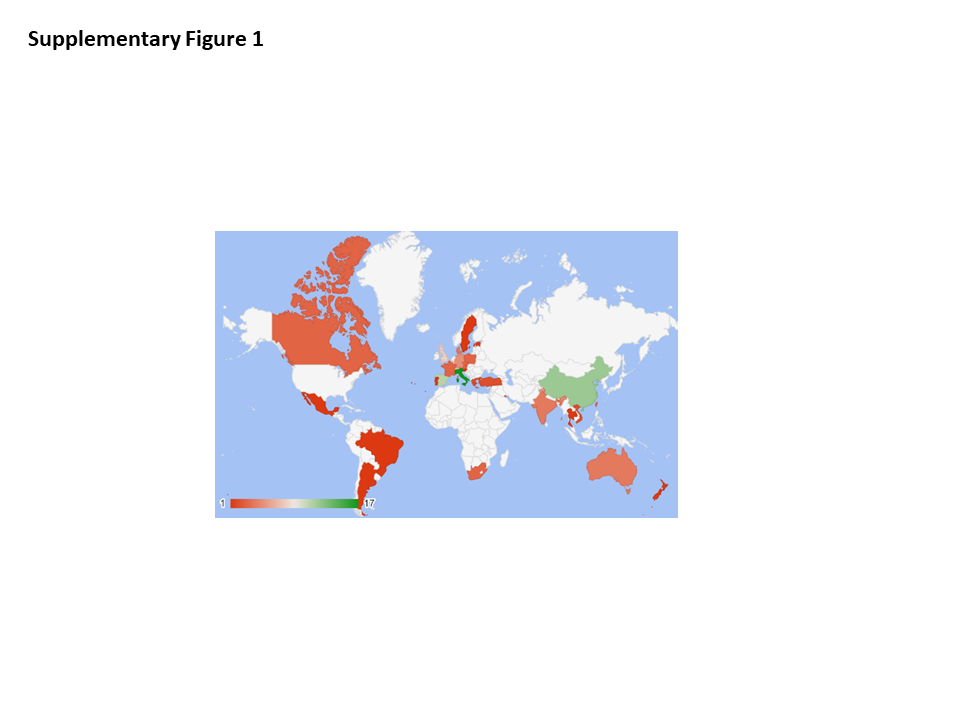

Supplement: Supplementary file 2 — High resolution image (TIF 114 kb) [file 428_2021_3093_MOESM1_ESM.tif]

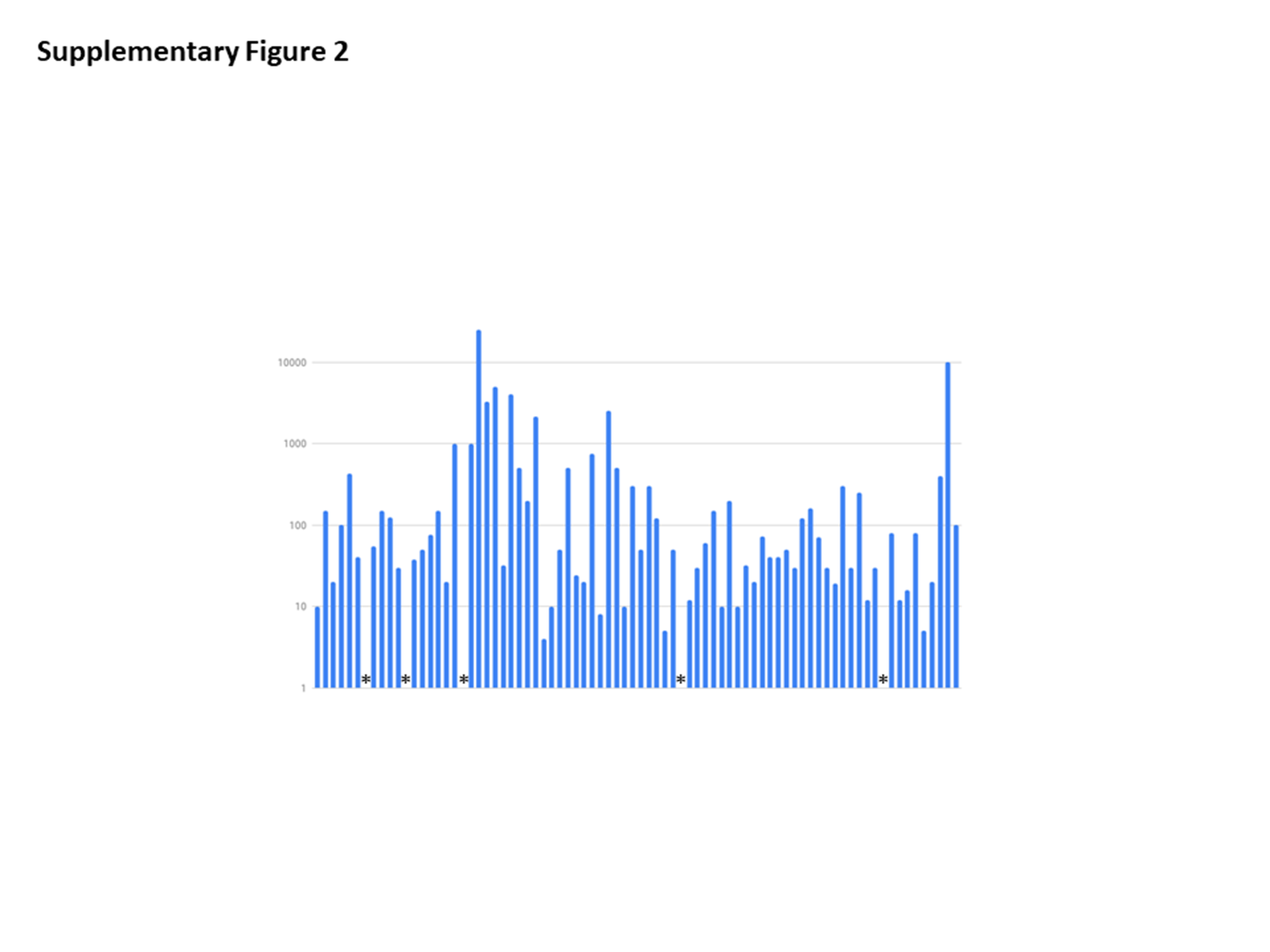

Supplement: Supplementary file 3 — Number of TMB tests performed by the responding laboratories. * = some laboratories did not disclose the number of TMB tests that they performed (PNG 162 kb) [file 428_2021_3093_Fig4_ESM.png]

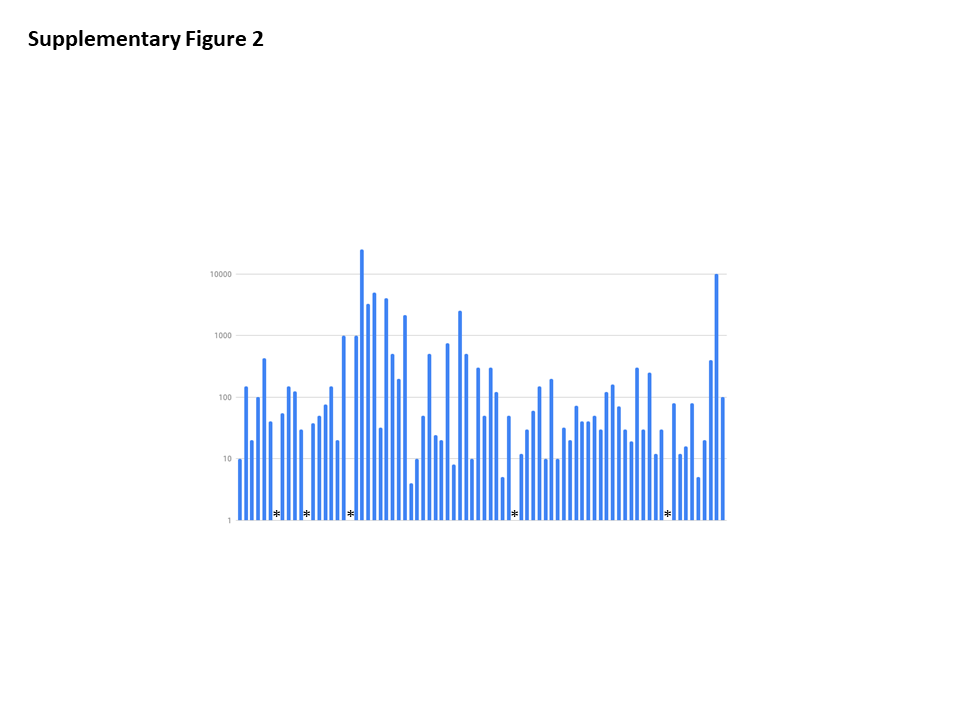

Supplement: Supplementary file 4 — High resolution image (TIF 124 kb) [file 428_2021_3093_MOESM2_ESM.tif]
